# The use of extracorporeal carbon dioxide removal to avoid intubation in patients failing non-invasive ventilation – a cost analysis

**DOI:** 10.1186/s12871-015-0139-0

**Published:** 2015-11-04

**Authors:** Stephan Braune, Hilmar Burchardi, Markus Engel, Axel Nierhaus, Henning Ebelt, Maria Metschke, Simone Rosseau, Stefan Kluge

**Affiliations:** Department of Intensive Care Medicine, University Medical Center Hamburg-Eppendorf, Martinistr. 52, 20246 Hamburg, Germany; Bovenden, Germany; Department of Cardiology and Intensive Care, Klinikum Bogenhausen, Munich, Germany; Department of Medicine III, University of Halle (Saale), Halle, Germany; Department of Internal Medicine, Infectious Diseases and Respiratory Medicine, Charité-Universitaetsmedizin Berlin, Berlin, Germany

**Keywords:** Extracorporeal, Carbon dioxide removal, Mechanical ventilation, Cost analysis, Treatment costs

## Abstract

**Background:**

To evaluate the economic implications of the pre-emptive use of extracorporeal carbon dioxide removal (ECCO_2_R) to avoid invasive mechanical ventilation (IMV) in patients with hypercapnic ventilatory insufficiency failing non-invasive ventilation (NIV).

**Methods:**

Retrospective ancillary cost analysis of data extracted from a recently published multicentre case–control-study (*n* = 42) on the use of arterio-venous ECCO_2_R to avoid IMV in patients with acute on chronic ventilatory failure. Cost calculations were based on average daily treatment costs for intensive care unit (ICU) and normal medical wards as well as on the specific costs of the ECCO_2_R system.

**Results:**

In the group treated with ECCO_2_R IMV was avoided in 90 % of cases and mean hospital length of stay (LOS) was shorter than in the matched control group treated with IMV (23.0 vs. 42.0 days). The overall average hospital treatment costs did not differ between the two groups (41.134 vs. 39.366 €, *p* = 0.8). A subgroup analysis of patients with chronic obstructive pulmonary disease (COPD) revealed significantly lower median ICU length of stay (11.0 vs. 35.0 days), hospital length of stay (17.5 vs. 51.5 days) and treatment costs for the ECCO_2_R group (19.610 vs. 46.552 €, *p* = 0.01).

**Conclusions:**

Additional costs for the use of arterio-venous ECCO_2_R to avoid IMV in patients with acute-on-chronic ventilatory insufficiency failing NIV may be offset by a cost reducing effect of a shorter length of ICU and hospital stay.

## Background

Over the last decades invasive mechanical ventilation (IMV) is increasingly applied to critically ill patients of a more and more ageing and comorbid population [1]. At the same time the negative side effects of IMV, such as ventilator-associated lung injury (VILI), ventilator-associated pneumonia (VAP), or ventilator-associated diaphragmatic dysfunction (VIDD) are recognised to contribute to morbidity and mortality [2–4]. In the last decade severe respiratory failure is increasingly treated with extracorporeal lung support, both for oxygenation (ECMO) and for carbon dioxide removal (ECCO_2_R) [5]. Recently, a new strategy of applying ECCO_2_R to avoid IMV and its side-effects in patients with hypercapnic acute-on-chronic ventilatory failure not responding to non-invasive ventilation (NIV) has been described [6–10]. The most common condition of acute-on-chronic ventilatory failure frequently leading to ICU admissions is an acute exacerbation of a chronic obstructive pulmonary disease (COPD) [11]. Once IMV is commenced after failure of NIV, especially patients with chronic pulmonary disease carry an increased risk of prolonged weaning and length of ICU stay and their overall prognosis deteriorates [12–18].

**Fig. 1:**
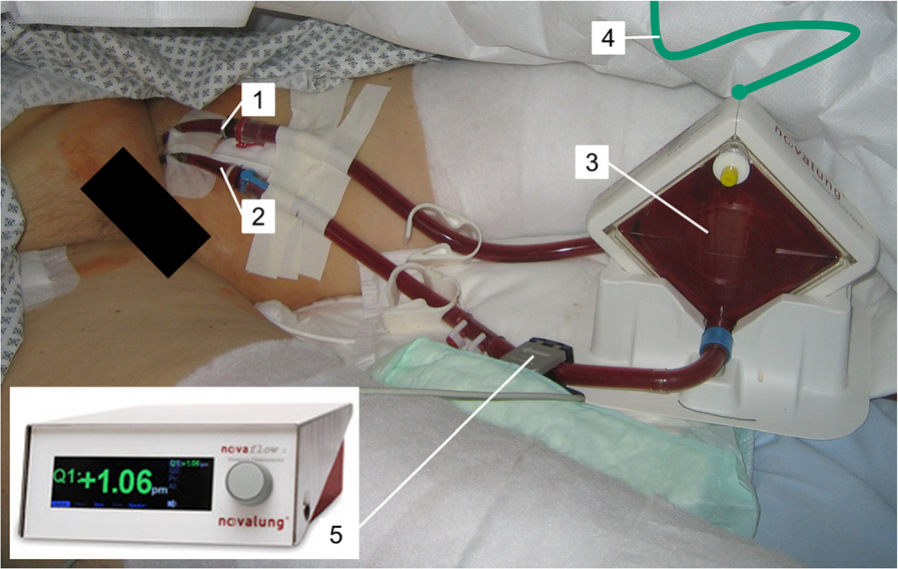
Clinical setup and components of the pumpless, arterio-venous ECCO_2_R circuit “interventional lung assist” (iLA®, Novalung GmbH, Heilbronn, Germany). The patient has given written consent for publication of this picture. 1 = arterial cannula, 2 = venous cannula, 3 = circuit including membrane for ECCO_2_R, 4 = sweep gas (O_2_), 5 = ultrasonic flow-meter

Our research group has recently published a matched case–control study on the feasibility and safety of ECCO_2_R in patients with NIV refractory acute on chronic hypercapnic ventilatory failure to avoid intubation [7]. The underlying chronic lung diseases of the study group are shown in Table 1. The extracorporeal device used was the so-called “interventional lung assist” (iLA®, Novalung GmbH, Heilbronn, Germany), a pumpless, arterio-venous circuit for ECCO_2_R (av-ECCO_2_R), which has been licensed in 2006 [19] and since then been applied worldwide to approximately 10.000 patients [20]. Figure 1 shows the clinical setup of the system (Fig 1). Further details are described elsewhere [19].

**Table 1:** Diagnoses of chronic respiratory diseases

Diagnosis	av-ECCO_2_R group	MV group
	*n* (%)	*n* (%)
Severe COPD	14 (66.7)	14 (66.7)
Cystic fibrosis	2 (9.5)	2 (9.5)
Pulmonary Graft-vs-Host-Disease	2 (9.5)	2 (9.5)
Pulmonary fibrosis	1 (4.8)	1 (4.8)
Bronchial asthma	1 (4.8)	1 (4.8)
Pneumonia post lung transplant	1 (4.8)	1 (4.8)

Intubation was avoided in 19 of 21 patients (90 %) treated with av-ECCO_2_R. Two patients in the av-ECCO_2_R group (9.5 %) had major bleeding complications. Ventilator-associated complications, such as VILI, VAP and VIDD, could not be recorded for methodological reasons. The median duration of av-ECCO_2_R support and mechanical ventilation in the av-ECCO_2_R group was 9 days (range 1–116) and the median duration of mechanical ventilation in the control group was 21 days (range 1–47; *p* = 0.944). Length of stay (LOS) in the ICU and in hospital was shorter in the av-ECCO_2_R group than that of the matched control group treated with invasive mechanical ventilation (15 vs. 30 days for LOS in ICU; *p* = 0.26, and 23 vs. 42 days for LOS in hospital; *p* = 0.05). Six month mortality was 33 % in both groups.

Despite its increasing use, nothing is known about the economic impact of the use of this complex and costly technology to avoid intubation and IMV. Thus the purpose of this ancillary economic analysis was to evaluate if the reduction in hospital length of stay also translated to reduced treatment costs.

## Methods

### Clinical study design and setting

This ancillary retrospective economic evaluation was undertaken using data from a recently published multicentre case–control-study on the use of an arterio-venous extracorporeal carbon dioxide removal device (av-ECCO_2_R) to avoid invasive mechanical ventilation in patients with acute on chronic respiratory insufficiency failing NIV [7]. The original study was conducted in four tertiary level hospitals in Germany (*Department of Intensive Care Medicine, University Medical Center Hamburg-Eppendorf; Department of Internal Medicine, Infectious Diseases and Respiratory Medicine, Charité-Universitaetsmedizin Berlin; Department of Medicine III, University of Halle (Saale*); *Department of Cardiology and Intensive Care, Klinikum Bogenhausen, Munich)*. The data collected in the case–control-study were routine clinical data from medical records. Because of the retrospective study design patient consent to participate in the study was not applicable. The institutional ethics committees of all four participating centres approved anonymised data collection and analyses.

### Patients and interventions

In 21 non-intubated patients with acute on chronic hypercapnic respiratory failure not responding to NIV and fulfilling criteria for intubation the iLA® device for ECCO_2_R had been commenced in order to avoid imminent intubation. All of these patients had potentially reversible respiratory failure, e.g. acute exacerbation of COPD, and endotracheal intubation was deemed to carry a substantial risk of secondary complications due to prolonged invasive mechanical ventilation. All av-ECCO_2_R circuits were inserted at the bed-side in the ICU by the attending intensivists. The study centers matched control patients had also been admitted with hypercapnic acute-on-chronic ventilatory failure but in contrast were intubated and placed on invasive mechanical ventilation after NIV failure. The matching criteria for the control group selection were: 1) underlying diagnosis, 2) age, 3) simplified acute physiology score (SAPS-2), and 4) arterial pH before ECCO_2_R or intubation [7]. All control patients underwent daily awakening and spontaneous breathing trials for ventilatory weaning according to the local protocol.

In addition to the economic evaluation of all 21 cases and their matched controls two subgroups were further analysed: The first subgroup was limited to those 17 ECCO_2_R cases and their corresponding controls, which subsequently were not lung transplanted. Because no control patient underwent lung transplantation costs were deemed not to be more comparable in this subgroup analysis. The second subgroup was further limited to all 12 cases with COPD not undergoing lung transplantation and their matched controls. The rationale for this was to evaluate a homogeneous patient group with the most common underlying diagnosis for acute on chronic ventilatory failure.

### Cost analysis

The costs were analysed from the hospital’s perspective and only direct treatment costs were evaluated [21]. All costs are expressed in 2013 Euro. Calculation of overall hospital costs for each case and control was based on average daily costs for treatment in the ICU and the medical normal ward. Average daily ICU costs were based on the results from the largest dataset on German national average daily ICU costs published by Moerer et al. [22]. Costs were derived from results of their subgroup analysis on patients on mechanical ventilation treated in tertiary level hospitals [22]. These costs included both variable and fixed costs. All costs derived in this study were 2003 Euros and were subsequently adjusted for the corresponding yearly inflation rates from 2004 until 2014, ranging from 0.3 to 2.6 % according to the German Federal Statistics Office [23].

Average daily costs for treatment in the normal medical ward were derived from the 2013 cost calculations of the study centres administrative data. These costs also included both variable and fixed costs. Applied average daily costs for ICU treatment and treatment on the medical ward are shown in Table 2.

**Table 2:** Values used for cost model

Item	Value (Euro)	Values for sensitivity analysis	
		Minimum value (Euro)	Maximum value (Euro)
Hospital costs			
• Daily costs for ICU	1115	697	1534
• Daily costs for normal medical ward	288	181	396
ECCO_2_R related costs			
• Single cannula	458	-	-
• Set with tubing and Novalung-Membrane	2335	-	-
• Daily rental costs for ultrasonic flow-meter	62/day	-	-

Abbreviations: ICU = intensive care unit, ECCO_2_R = Extracorporeal carbon dioxide removal

In addition, specific costs for av-ECCO_2_R treatment were calculated for each case based on the actual utilisation of consumables (cannulas and membranes) and length of treatment (daily rental costs for ultrasonic flow meter) (Fig. 1). Costs for consumables and daily rental costs for the flow meter were based on the manufacturer’s official list prices for Germany in 2013. All cost items for av-ECCO_2_R and their prices are listed in Table 2. Any specific costs for mechanical ventilation were subsumed in the average daily ICU costs as derived in the original study by Moerer et al. [22].

ICU treatment costs for each case and control were calculated by multiplying the average daily ICU costs by the number of days spent in the ICU. In the case group specific costs for av-ECCO_2_R treatment were added to the ICU costs. Costs for treatment on the normal wards were calculated by multiplying average daily costs for a normal medical ward with the number of days actually spent on the normal ward after ICU discharge until hospital discharge or death. Finally, treatment costs for ICU and the normal ward were added to estimate the total hospital treatment costs.

To test the robustness of these cost estimates and to assess the impact of variations of daily ICU and normal ward costs, sensitivity analyses were performed. Average daily ICU costs calculated by Moerer et al. [22] were varied by applying the corresponding upper and lower standard deviations. Average daily costs for normal medical wards were varied by 30 %. Upper and lower margins for average daily treatment costs are presented in Table 2.

Diagnosis-related groups (DRG) and specific reimbursement plans for mechanical ventilation and extracorporeal lung support were not analysed.

### Statistical methods

Results are presented as absolute numbers and percentages, as means with standard deviations for continuous variables if normally distributed, and as medians with ranges if not normally distributed. Comparisons between the two groups were performed using the *t*-test or the Mann–Whitney *U* test depending on the data distribution. Two-sided *p* < 0.05 values were considered significant. The software used was SPSS® (version 20.0, SPSS Inc., Chicago, IL, USA).

## Results

Median hospital length of stay (LOS) was shorter in the av-ECCO_2_R group than in the matched control group treated with IMV (23 vs. 42 days, *p* = 0.05). The overall mean hospital treatment costs did not differ significantly between the two groups (41.134 vs. 39.366 €, *p* = 0.80). A sensitivity analysis did not reveal any significant differences when applying higher margins (53.689 vs. 54.155 €, *p* = 0.98) or lower margins (28.616 vs. 24.629 €, *p* = 0.60). Average ICU costs of the av-ECCO_2_R and the control group were 38.459 € (93.5 % of all hospital costs) and 33.290 € (84.6 % of all hospital costs), respectively. Average costs for av-ECCO_2_R, included in the ICU costs of the case group, were 7.717 € (18.8 %).

In the subgroup of 17 cases and controls without lung transplantation 6-months mortality rates were 41.2 and 35.3 % respectively (*p* = 1.0). The median hospital LOS and ICU-LOS were 22 vs. 42 days (*p* = 0.41) and 13 vs. 30 days (*p* = 0.23). Overall mean hospital treatment costs did not differ significantly between av-ECCO_2_R cases and controls (33.843 vs. 39.731 €; *p* = 0.64) and a sensitivity analysis did not change these non-significant differences.

A subgroup analysis of each 12 non-lung-transplanted COPD cases and their matched COPD controls revealed no significant differences in 6-months mortality (33.3 % vs. 25.0 %; *p* = 1.0). Hospital LOS (17.5 vs. 51.0 days; *p* = 0.01) and ICU-LOS (11.0 vs. 35.0 days; *p* = 0.01) were significantly shorter in the av-ECCO_2_R group. Mean hospital treatment costs were also significantly lower in the ECCO_2_R group compared to the control group (19.610 vs. 46.552 €, *p* = 0.01; 95 % confidence interval (95%CI) of absolute cost difference 5.769–48.113 €). A sensitivity analysis also showed significant differences. Detailed results of all groups and subgroups are presented in Table 3.

**Table 3:** Comparisons of LOS and costs with sensitivity analysis and subgroup analysis

All study patients			
Variable	ECCO_2_R	Control	*p* value
Days [range] or Euro [SD]	(*n* = 21)	(*n* = 21)	
Median length of stay in ICU	15 [4–137]	30 [4–66]	0.58
Median length of stay in hospital	23 [4–137]	42 [4–248]	0.05
Median duration of invasive MV	0 [0–47]	21 [4–47]	<0.001
Mean costs for ECCO_2_R	7717 [7835]	-	-
Mean total ICU costs	38460 [43878]	33291 [22572]	0.63
Mean total hospital costs	41134 [43005]	39366 [29903]	0.88
Mean hospital costs with maximal values	53689 [56406]	54155 [41133]	0.98
Mean hospital costs with minimal values	28616 [18721]	24629 [18721]	0.61
Subgroup analysis after exclusion of patients with lung transplantation			
Variable	ECCO_2_R	Control	*p* value
Days [range] or Euro [SD]	(*n* = 17)	(*n* = 17)	
Median length of stay in ICU	13 [4–137]	30 [4–66]	0.41
Median length of stay in hospital	22 [4–137]	42 [4–248]	0.23
Median duration of invasive MV	0 [0–47]	21 [1–47]	0.001
Mean costs for ECCO_2_R	5972 [6522]	-	-
Mean total ICU costs	24989 [35273]	33581 [23656]	0.41
Mean total hospital costs	33843 [40866]	39731 [31507]	0.64
Mean hospital costs with maximal values	44314 [53924]	54656 [43340]	0.54
Mean hospital costs with minimal values	23405 [27856]	24857 [19727]	0.86
Subgroup analysis of patients with COPD			
Variable	ECCO_2_R	Control	*p* value
Days [range] or Euro [SD]	(*n* = 12)	(*n* = 12)	
Median length of stay in ICU	11 [4–23]	35 [4–66]	0.004
Median length of stay in hospital	17 [4–43]	51 [4–248]	0.04
Median duration of invasive MV	0 [0–22]	27 [4–47]	0.001
Mean costs for ECCO_2_R	4472 [1269]	-	-
Mean total ICU costs	13194 [4611]	39304 [25163]	0.004
Mean total hospital costs	19610 [7509]	46552 [34558]	0.02
Mean hospital costs with maximal values	25298 [10115]	64040 [47536]	0.01
Mean hospital costs with minimal values	13942 [4938]	29124 [21641]	0.03

*Abbreviations*: *LOS* length of stay, *SD* standard deviation, *COPD* chronic obstructive pulmonary disease, *ICU* intensive care unit, *MV* mechanical ventilation, *ECCO*_*2*_ *R* Extracorporeal carbon dioxide removal

## Discussion

This study is the first economic evaluation to compare the costs of an av-ECCO_2_R strategy to avoid IMV with costs for a conventional strategy of IMV after NIV failure in patients with acute-on-chronic ventilatory insufficiency. The overall hospital costs did not differ significantly between the av-ECCO_2_R group and the matched control group (53.689 vs. 54.155 €) despite additional average costs of 7717 € per av-ECCO_2_R treatment. This did not change after excluding four ECCO_2_R patients who underwent lung transplantation and their controls. Thus, in this study the extra costs for av-ECCO_2_R treatment were offset by lower hospital costs due to shorter average hospital length of stay in the av-ECCO_2_R group. A short length of stay in the ICU may per se result in a reduction of treatment costs, as it has previously been shown that length of ICU stay explains approximately 85 to 90 % of interpatient variation in hospital costs [24].

Analysis of all COPD patients, the biggest and most homogenous diagnostic subgroup, revealed significantly lower overall hospital treatment costs in the av-ECCO_2_R group (19.610 vs. 46.552 €) suggesting that ECCO_2_R to avoid intubation in patients with AECOPD and NIV failure may lead to a reduction of overall treatment costs. The potential economical relevance of this hypothesis is augmented by the fact that COPD has a high prevalence projected to be the fifth leading burden of disease worldwide by the year 2020 [11, 25].

Micro-costing was used for cost calculation of material consumption for av-ECCO_2_R. Itemised cost evaluation of all other treatment costs was not feasible due to the retrospective study design. Instead, further cost analysis was performed by means of macro-costing. Average daily costs for ICU treatment used for cost analysis in this study were based on a previous study by Moerer et al. who calculated the average daily ICU costs in Germany by cost analyses of 51 German ICUs [22]. Their calculations included both variable and fixed costs as were costs for IMV. However, many health economic studies have demonstrated that fixed costs, especially staffing costs, account for up to 65 % of all treatment costs [22, 26–29]. Thus variation of variable costs probably has a smaller effect of overall treatment costs. Moreover, sensitivity analysis did not change the results suggesting a degree of robustness of the results.

The rationale for applying the above mentioned data on average ICU costs also to ECCO_2_R patients despite the fact that the majority this group were not intubated and mechanically ventilated was, that from the experience of the participating study centres staff resource utilisation for management of invasive MV and av-ECCO_2_R with or without non-invasive ventilation is comparable. In addition, costs for material consumption from MV were proposed to be relatively low and not significantly contributing to the overall treatment costs. Costs for treatment of side effects of av-ECCO_2_R observed in the original case–control study, such as bleeding and vascular surgery in one case, were not itemised separately. These costs were included in the average daily ICU costs as were treatment costs in the control group for side effects caused by invasive MV, such as treatment of ventilator-induced pneumonia. There is evidence that costs for patients on MV are significantly higher than costs for non-ventilated patients in ICU [22, 30, 31] and that COPD patients on invasive mechanical ventilation have a longer stay in ICU and require higher treatment efforts than non-COPD patients on MV [32]. These data may further justify subsuming costs for side effects of av-ECCO_2_R into average daily ICU costs and may even suggest that a potential cost saving effect of avoiding MV with its complications and associated costs by applying av-ECCO_2_R may have been underestimated. On the other hand, additional costs for complications caused by av-ECCO_2_R may also be associated with significant costs. The incidence of major, potentially costly complications was low in the av-ECCO_2_R group (9.5 %), but may have been underestimated because of low case numbers. Excluding 4 case-controls where the av-ECCO_2_R cases subsequently underwent lung transplantation with potentially higher costs did not change the results.

Since there are no valid national data on average daily costs for treatment of patients on normal wards in Germany who have previously been in ICU with respiratory failure, estimating these costs was based on the study centres local average cost calculations. These locally derived calculations may be less generalizable. However, as only less than 25 % of the total hospital costs were attributable to treatment on the normal ward, this potential error may be negligible.

This relationship is in line with findings of other studies that have shown that length of ICU stay was the main determinant of overall hospital cost variations [24, 33]. Again, sensitivity analysis had no significant effect on the results of this cost comparison.

Since cost calculations for ECCO_2_R were based on the pumpless arterio-venous ECCO_2_R device applied in the original study, these results cannot directly be applied to veno-venous, pump-driven devices for ECCO_2_R since their technology is more complex and extra cost apply for more expensive circuits and consoles. In addition, the clinical characteristics of patients and their controls of this study are quite specific as they had actually *failed* NIV and *all* patients in the control group had been intubated. Therefore they differ from the population of a recently published case–control-study in which patients *at risk of* NIV failure where treated with ECCO_2_R to avoid intubation [10]. In this study 25 patients treated with NIV and in addition a veno-venous, pump-driven ECCO_2_R device revealed a significantly lower intubation rate in comparison to their 21 controls treated with NIV alone (12 % vs. 33 %). The LOS in ICU and hospital did not differ significantly between the two groups (8 vs. 12 and 24 vs. 22, respectively).

Further, this cost analysis did not take into account the health care provider’s perspective on reimbursement plans, because aspects of diagnosis-related groups (DRG) or specific reimbursements for costly treatments and procedures such as invasive mechanical ventilation and extracorporeal lung support were not addressed. The rational for this was that reimbursement plans are individually negotiated between health care providers and healthcare payers on a local, regional and/or national level. Reimbursements do not necessarily represent factual costs and are highly variable between health care systems and over time [31, 34]. In fact, successfully avoiding intubation and MV by means of ECCO_2_R paradoxically may lead to an economic disadvantage for a specific health care provider if “lost” reimbursement rates for (prolonged) MV are not compensated for by reimbursement rates for ECCO_2_R treatment. Therefore further prospective and larger clinical and economic studies on strategies to avoid MV by means of ECCO_2_R are warranted to provide more evidence for rational decision making both in clinical practice and on a health care system level.

## Conclusion

The additional costs of the use of ECCO_2_R to avoid IMV in NIV failure in a mixed group of patients with acute-on-chronic ventilatory insufficiency may be offset by a potential cost reduction through a shorter length of hospital and ICU stay. Moreover, in patients with acute exacerbation of COPD this novel treatment strategy may not only hold clinical advantages, but may even reduce overall resource utilization and treatment costs. However, the preliminary and hypothesis-generating results of this pilot study must be validated in larger, prospective, multicentre, high quality randomised clinical trials not only evaluating clinical benefits but also paralleled by evaluation of cost-effectiveness applying a micro-costing approach.

### Key message

Additional costs associated with the use of arterio-venous extracorporeal carbon dioxide removal to avoid invasive mechanical ventilation in patients with acute-on-chronic ventilatory insufficiency failing non-invasive ventilation may be offset by a potential cost reduction through a shorter length of ICU and hospital stay.

## Abbreviations

av-ECCO_2_R: arterio-venous extracorporeal carbon dioxide removal
COPD: Chronic obstructive pulmonary disease
DRG: Diagnosis-related group
ECCO_2_R: Extracorporeal carbon dioxide removal
ECMO: Extracorporeal membrane oxygenation
ICU: Intensive care unit
IMV: Invasive mechanical ventilation
LOS: Length of stay
NIV: Non-invasive ventilation
VAP: Ventilator-associated pneumonia
VILI: Ventilator-induced lung injury
VIDD: Ventilator induced diaphragmatic dysfunction

## References

[1] Kahn JM, Le T, Angus DC, Cox CE, Hough CL, White DB, Yende S, Carson SS (2015). The epidemiology of chronic critical illness in the United States. Crit Care Med.

[2] Slutsky AS, Ranieri VM (2013). Ventilator-induced lung injury. N Engl J Med.

[3] Jaber S, Jung B, Matecki S, Petrof BJ (2011). Clinical review: ventilator-induced diaphragmatic dysfunction - human studies confirm animal model findings!. Crit Care.

[4] Timsit JF, Zahar JR, Chevret S (2011). Attributable mortality of ventilator-associated pneumonia. Curr Opin Crit Care.

[5] Agerstrand CL, Bacchetta MD, Brodie D (2014). ECMO for adult respiratory failure: current use and evolving applications. ASAIO J.

[6] Terragni PP, Birocco A, Faggiano C, Ranieri VM (2010). Extracorporeal CO2 removal. Contrib Nephrol.

[7] Kluge S, Braune SA, Engel M, Nierhaus A, Frings D, Ebelt H, Uhrig A, Metschke M, Wegscheider K, Suttorp N, Rousseau S (2012). Avoiding invasive mechanical ventilation by extracorporeal carbon dioxide removal in patients failing noninvasive ventilation. Intensive Care Med.

[8] Burki NK, Mani RK, Herth FJ, Schmidt W, Teschler H, Bonin F, Becker H, Randerath WJ, Stieglitz S, Hagmeyer L, Priegnitz C, Pfeifer M, Blaas SH, Putensen C, Theuerkauf N, Quintel M, Moerer O (2013). A novel extracorporeal CO(2) removal system: results of a pilot study of hypercapnic respiratory failure in patients with COPD. Chest.

[9] Crotti S, Lissoni A, Tubiolo D, Azzari S, Tarsia P, Caspani L, Gattinoni L (2012). Artificial lung as an alternative to mechanical ventilation in COPD exacerbation. Eur Respir J.

[10] Del Sorbo SL, Pisani L, Filippini C, Fanelli V, Fasano L, Terragni P, Dell’Amore A, Urbino R, Mascia L, Evangelista A, Antro C, D’Amato R, Sucre MJ, Simonetti U, Persico P, Nava S, Ranieri VM (2015). Extracorporeal Co2 removal in hypercapnic patients at risk of noninvasive ventilation failure: a matched cohort study with historical control. Crit Care Med.

[11] Perera PN, Armstrong EP, Sherrill DL, Skrepnek GH (2012). Acute exacerbations of COPD in the United States: inpatient burden and predictors of costs and mortality. COPD.

[12] Nevins ML, Epstein SK (2001). Predictors of outcome for patients with COPD requiring invasive mechanical ventilation. Chest.

[13] Esteban A, Anzueto A, Frutos F, Alia I, Brochard L, Stewart TE, Benito S, Epstein SK, Apezteguia C, Nightingale P, Arroliga AC, Tobin MJ (2002). Characteristics and outcomes in adult patients receiving mechanical ventilation: a 28-day international study. JAMA.

[14] Ucgun I, Metintas M, Moral H, Alatas F, Yildirim H, Erginel S (2006). Predictors of hospital outcome and intubation in COPD patients admitted to the respiratory ICU for acute hypercapnic respiratory failure. Respir Med.

[15] Texereau J, Jamal D, Choukroun G, Burgel PR, Diehl JL, Rabbat A, Loirat P, Parrot A, Duguet A, Coste J, Dusser D, Hubert D, Mira JP (2006). Determinants of mortality for adults with cystic fibrosis admitted in Intensive Care Unit: a multicenter study. Respir Res.

[16] Menzies R, Gibbons W, Goldberg P (1989). Determinants of weaning and survival among patients with COPD who require mechanical ventilation for acute respiratory failure. Chest.

[17] Combes A, Costa MA, Trouillet JL, Baudot J, Mokhtari M, Gibert C, Chastre J (2003). Morbidity, mortality, and quality-of-life outcomes of patients requiring > or = 14 days of mechanical ventilation. Crit Care Med.

[18] Pfeifer M (2013). Chronic critically ill patients from a pneumological perspective. Med Klin Intensivmed Notfmed.

[19] Bein T, Weber F, Philipp A, Prasser C, Pfeifer M, Schmid FX, Butz B, Birnbaum D, Taeger K, Schlitt HJ (2006). A new pumpless extracorporeal interventional lung assist in critical hypoxemia/hypercapnia. Crit Care Med.

[20] Novalung GmbH (2014). Annual Business Report.

[21] Jegers M, Edbrooke DL, Hibbert CL, Chalfin DB, Burchardi H (2002). Definitions and methods of cost assessment: an intensivist’s guide. ESICM section on health research and outcome working group on cost effectiveness. Intensive Care Med.

[22] Moerer O, Plock E, Mgbor U, Schmid A, Schneider H, Wischnewsky MB, Burchardi H (2007). A German national prevalence study on the cost of intensive care: an evaluation from 51 intensive care units. Crit Care.

[23] Consumer price index and inflation rates. German Federal Statistical Office. https://www.destatis.de/EN/Homepage.html. Accessed 27 June 2015

[24] Rapoport J, Teres D, Zhao Y, Lemeshow S (2003). Length of stay data as a guide to hospital economic performance for ICU patients. Med Care.

[25] Mannino DM, Buist AS (2007). Global burden of COPD: risk factors, prevalence, and future trends. Lancet.

[26] Parviainen I, Herranen A, Holm A, Uusaro A, Ruokonen E (2004). Results and costs of intensive care in a tertiary university hospital from 1996–2000. Acta Anaesthesiol Scand.

[27] Edbrooke DL, Minelli C, Mills GH, Iapichino G, Pezzi A, Corbella D, Jacobs P, Lippert A, Wiis J, Pesenti A, Patroniti N, Pirracchio R, Payen D, Gurman G, Bakker J, Kesecioglu J, Hargreaves C, Cohen SL, Baras M, Artigas A, Sprung CL (2011). Implications of ICU triage decisions on patient mortality: a cost-effectiveness analysis. Crit Care.

[28] Edbrooke D, Hibbert C, Ridley S, Long T, Dickie H (1999). The development of a method for comparative costing of individual intensive care units. The Intensive Care Working Group on Costing. Anaesthesia.

[29] Flaatten H, Kvale R (2003). Cost of intensive care in a Norwegian University hospital 1997–1999. Crit Care.

[30] Dasta JF, McLaughlin TP, Mody SH, Piech CT (2005). Daily cost of an intensive care unit day: the contribution of mechanical ventilation. Crit Care Med.

[31] Heyland DK, Gafni A, Kernerman P, Keenan S, Chalfin D (1999). How to use the results of an economic evaluation. Crit Care Med.

[32] Makris D, Desrousseaux B, Zakynthinos E, Durocher A, Nseir S (2011). The impact of COPD on ICU mortality in patients with ventilator-associated pneumonia. Respir Med.

[33] Norris C, Jacobs P, Rapoport J, Hamilton S (1995). ICU and non-ICU cost per day. Can J Anaesth.

[34] Gyldmark M (1995). A review of cost studies of intensive care units: problems with the cost concept. Crit Care Med.

